# Testing an unusual *in vivo* vessel network model: a method to study angiogenesis in the colonial tunicate *Botryllus schlosseri*

**DOI:** 10.1038/srep06460

**Published:** 2014-09-24

**Authors:** Fabio Gasparini, Federico Caicci, Francesca Rigon, Giovanna Zaniolo, Lucia Manni

**Affiliations:** 1 Dipartimento di Biologia, Università degli Studi di Padova, Via Ugo Bassi 58/B, 35131, Padova, Italy; 2 CORIT-Consortium for Research in Organ Transplantation, Legnaro, 35020 Padova, Italy

## Abstract

Tunicates are the closest relatives to vertebrates and include the only chordate species able to reproduce both sexually and asexually. The colonial tunicate *Botryllus schlosseri* is embedded in a transparent extracellular matrix (the tunic) containing the colonial circulatory system (CCS). The latter is a network of vessels external to zooids, limited by a simple, flat epithelium that originated from the epidermis. The CCS propagates and regenerates by remodelling and extending the vessel network through the mechanism of sprouting, which typically characterises vertebrate angiogenesis. In exploiting the characteristics of *B. schlosseri* as a laboratory model, we present a new experimental and analysis method based on the ability to obtain genetically identical subclones representing paired samples for the appropriate quantitative outcome statistical analysis. The method, tested using human VEGF and EGF to induce angiogenesis, shows that the CCS provides a useful *in vivo* vessel network model for testing the effects of specific injected solutes on vessel dynamics. These results show the potentiality of *B. schlosseri* CCS as an effective complementary model for *in vivo* studies on angiogenesis and anticancer therapy. We discuss this potentiality, taking into consideration the origin, nature, and roles of the cellular and molecular agents involved in CCS growth.

The process of blood vessel formation is essential for a wide variety of human physiological and pathological processes^1^,^2^,^3^. Commonly, it occurs during vertebrate embryogenesis and occasionally in adult regenerative-renewal conditions, such as in liver regeneration, the proliferative phase of the mammalian uterine cycle, or in tumour vascularisation^2^,^4^,^5^. Understanding the mechanisms of its regulation should provide novel insights for clinical manipulations of pathological conditions. For example, vessel formation is required to supply solid tumours with nutrients from the blood and allows for tumour survival, growth, and metastasis^6^. Thus, the study of angiogenesis, the main mechanism of new vessel formation^7^, attracts many researchers. Special emphasis is placed on the development of new angiogenic-related drugs, as well as therapies that target tumours, such as angiogenesis inhibition or drug delivery systems^8^,^9^,^10^. Searching for appropriate animal models for this type of research is an appealing field and may help develop new experimental platforms for the essential link between bench cell-based experiments and the bedside^11^,^12^. Among these models, there are definite benefits in using small, simple, whole organisms that are easy to manipulate and evolutionarily close to vertebrates.

The colonial chordate *Botryllus schlosseri* provides a remarkable resource for the study of angiogenesis. The mechanism of blood vessel formation has been elucidated, and interesting correlations with that of vertebrates have been found^13^,^14^. Studies on this species have contributed to the understanding of angiogenesis evolution and the involved signalling cascades^15^,^16^.

*B. schlosseri* belongs to the tunicates, which are considered the closest living relatives to vertebrates and includes the only chordate species able to reproduce both sexually and asexually. Indeed, *B. schlosseri* possesses the two reproductive modes: sexual reproduction, commonly used for dispersion of individuals with re-assorted new genomes, and asexual reproduction (also called blastogenesis or budding) for formation of large colonies containing numerous individuals (zooids) (Fig. 1)^17^,^18^,^19^. *B. schlosseri* has internal fertilisation and is ovoviviparous. Mature colonies release swimming tadpole larvae, which adhere to the substrate and metamorphose within 36–48 hr into sessile functional oozooids approximately 0.5 mm in length. A larva is the founder of a new colony. It bears one bud, which grows on one side of the newly settled oozooid and forms the first adult blastozooid (*i.e.*, zooid derived from budding). It is then able to produce several lateral buds. Each colony is composed of numerous individuals organised in star-shaped systems of up to a dozen filtering zooids (Fig. 1, Fig. 2 A). In each system the clonal blastozooids are arranged with their posterior regions around a common cloacal excurrent siphon. Conversely, the oral incurrent siphon opens individually on the anterior part of each zooid (Fig. 1).

In each colony, three blastogenetic generations coexist: the filtering adults, their buds (primary buds) and the budlets (secondary buds) of the last generation. All the zooids of a blastogenetic generation progressively reach the same developmental stage, and their developmental progression is synchronised with the other two generations. Moreover, one to five buds develop from a specific stage of the parent^17^,^20^. This results in a colonial life cycle characterised by the presence, time after time, of zooids in three specific stages. Deviations from the synchronised condition are symptoms of colonial distress^21^. Cyclic changes in blastogenetic generations (termed “take-over”) occur weekly. All the adults regress, while their buds begin to filter, thus becoming new adults, and a new generation of budlets is produced. The blastogenetic cycle starts with the opening of the siphons of new adult zooids and ends with the conclusion of the take-over phase when the next blastogenetic generation reaches functional maturity. This process, in which buds and budlets gradually grow, takes one week at 18°C^20^.

Zooids are embedded in a thin transparent tunic, the typical extracellular matrix of tunicates which form, with its scattered cells, a complex connective tissue outside the epidermis. Tunic contains cellulose cross linked with proteins^22^,^23^. In this species the tunic contains also the colonial circulatory system (CCS). This is an intricate network of anastomosed vessels that is external to the zooids and is limited by a simple, flat epithelium that originated by epidermal extrusion^14^,^24^.

As shown in Figs. 1 and 2 B, the main CCS components are (1) the marginal vessel running around the periphery of the colony, (2) the radial vessels connecting each zooid to the CCS network, and (3) the ampullae, which are contractile blind-ended structures that are mainly located at the periphery of the colony. During the growth of a colony, the CCS propagates remodelling and extending the vessel network through the mechanism of sprouting, *i.e.*, the same mechanism that typically characterises vertebrate angiogenesis^14^. Moreover, antibodies against human Epidermal Growth Factor (EGF), Fibroblast Growth Factor 2 (FGF2), and Vascular Endothelial Growth Factor (VEGF) and their receptors specifically recognise angiogenic sites, indicating that *B. schlosseri* angiogenic pathways correspond to those of vertebrates^13^,^14^.

The analysis of experimentally ablated vascular systems in *B. schlosseri* colonies has shown that vessel regeneration also occurs by sprouting^13^. Moreover, it has been found that the regeneration of the *B. schlosseri* CCS is under the control of the major mediator of new blood vessel formation in vertebrates, the VEGF^25^,^26^.

*B. schlosseri* colonies can be cultured adhering to glass slides in the laboratory in tanks with seawater, and experimental procedures such as CCS ablation and microinjection into the vasculature can be easily performed. The CCS development and regeneration occur according to a bi-dimensional propagation and can be followed *in vivo* under the microscope due to transparency of the tunic (Fig. 2 C–H). In addition, the systems of a colony can be experimentally subdivided, providing subclones possessing the same genotype. All of these characteristics (as evidenced in other studies^13^,^27^,^28^), in addition to its key phylogenetic position, indicate that the *B. schlosseri* CCS can be a useful and highly accessible experimental model for *in vivo* angiogenic studies that will contribute to the understanding of involved mechanisms and their evolution in chordates^13^,^14^,^25^,^26^.

In this study, using the human endogenous pro-angiogenic factors VEGF and EGF^29^,^30^ to impact angiogenesis, we describe a new experimental and data analysis protocol for testing the effects of injected solutes into the *B. schlosseri* CCS to determine if this model could be used for more extensive studies regarding angiogenic-related molecules used in humans.

## Results

### *In vivo* approach and observations of tunic and CCS regeneration

In our experiments, pairs of subclones (biological replicas), each consisting of two small colonies composed of one to three systems, were prepared by fragmenting a same-parental colony. A surgical procedure was then performed to induce CCS regeneration as previously described^13^. Briefly, the tunic and the included network of vessels were removed from around four to five zooids. The CCS was cut at the lateral boundaries of the chosen surgery area (lateral cut edges) and around the zooids (proximal cut edge); then it was excised, and the injured area was removed (Fig. 3 A).

Nineteen pairs of subclones that were operated upon, were then injected in parallel with human VEGF and phosphate buffer saline (PBS) as a control. Twelve pairs of subclones that were operated upon, were then injected in parallel with human EGF and PBS as a control. One microliter of VEGF or EGF at a concentration of 500 ng/μl was chosen as the initial injection concentration. This dose, which is relatively higher with respect the ones mainly used in mammals^31^,^32^, was chosen taking in consideration the ability of *B. schlosseri* to maintain colony homeostasis in experimental conditions^20^. Using this concentration, we analysed the general health of the colonies injected with the protein of interest, with respect to their own controls as a toxicity indicator (see next paragraph) and then applied the statistical analysis, to verify the effect of the injected protein on the CCS. Applying our method, we verified that the injected dose of both VEGF and EGF was adequate to significantly induce angiogenesis. The chosen concentration therefore was enough to describe the goodness of the experimental and analysis method and no other experiments were done to test if these factors impacted strongly at further concentrations on *B. schlosseri* (see next section of the Results and the Discussion). No changes in this protocol has been done during the study. The following parameters have been used to check the healthy condition of colonies: i) the progression of the zooid stage in relationship to the developmental synchronisation between blastogenetic generations, ii) the fluency of blood circulation, iii) the vitality of zooids evaluated on the basis of their response to mechanical stimulation with the tip of a needle, iv) the state of aggregation of zooids in well-organised systems, and v) the condition of the tunic (transparency, blood vessels supply, presence/absence of necrotic areas).

The experimental ablation produced a series of early reactions in the colony comparable to those previously described during observation of CCS regeneration^13^. These regarded tunic, vessel and ampullae formation and changes in blood flow circulation. Briefly, the effects were: i) blood leakage at the cut level that stopped within a few seconds, ii) stopped blood flow and zooid contraction, a condition that continued for approximately 1 h, after which iii) circulation was restored, and zooid relaxation was achieved. The injection usually caused a shrinkage of each involved ampulla with a slight leakage of blood when the glass micropipette was removed from the epithelium, leaving a small wound that healed rapidly by clotting of the blood cells. All colonies appeared healthy in the following days, in that the developmental timing and vitality of blastozooids, the state of aggregation of adults in systems (each one with a well-formed cloacal siphon), the synchronisation between blastogenetic generations, tunic transparency and its blood irroration all indicated that the microinjections had not perturbed the colonies^20^,^21^.

**Figure 2:**
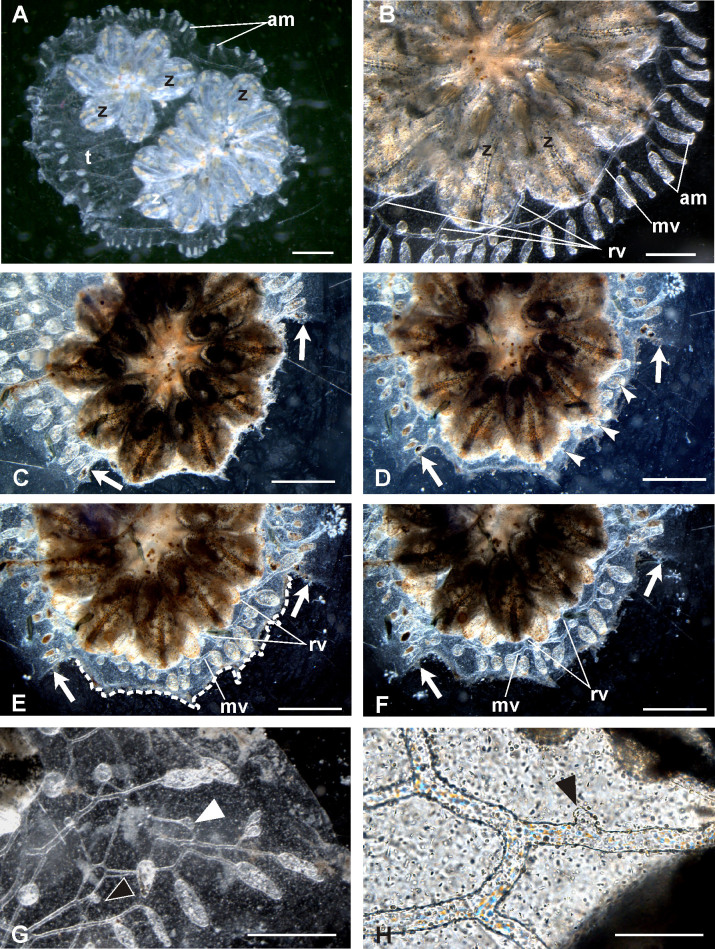
Ventral views of *Botryllus schlosseri* colonies grown on glass slides. (A) A colony formed by two star shaped systems of six and twelve adult zooids (z), respectively, is embedded in the common transparent tunic (t). The crown of ampullae (am) is visible at the periphery of the colony. (B) Peripheral region of a colony in which the main components of the colonial circulatory system (CCS) are recognisable: the marginal vessel (mv), some radial vessels (rv) and some ampullae (am). (C) Detail of an operated colony, two hours after the ablation of the CCS in front of four adult zooids. Arrows indicate the lateral cut edges. (D) The same colony of (C), injected with PBS, one day after ablation. The thin line of new tunic, penetrated by vessel stumps and small ampullae (arrowheads), covers the previously exposed zooids. (E–F) The same colony showed in (C), two and three days after ablation. Both lateral (arrows) and proximal (dotted line in (E)) cut edges moved to reach the tunic profile external to the ablated region. In the regenerated tunic, the marginal vessel (mv) and the crown of ampullae appear almost fully regenerated in (F) with respect to the day before (E). Similarly, more regenerated radial vessels are visible in (F) with respect to (E). (G, H) Magnifications of CCS regenerated region in colonies. Elongating new vessels (white arrowhead) and sprouts (black arrowheads) are well visible. Scale bars: 1 mm in (A, C–F); 500 μm in (B, F); 100 μm in (G). Images were organized with CorelDRAW X4 (Corel Corporation).

**Figure 3:**
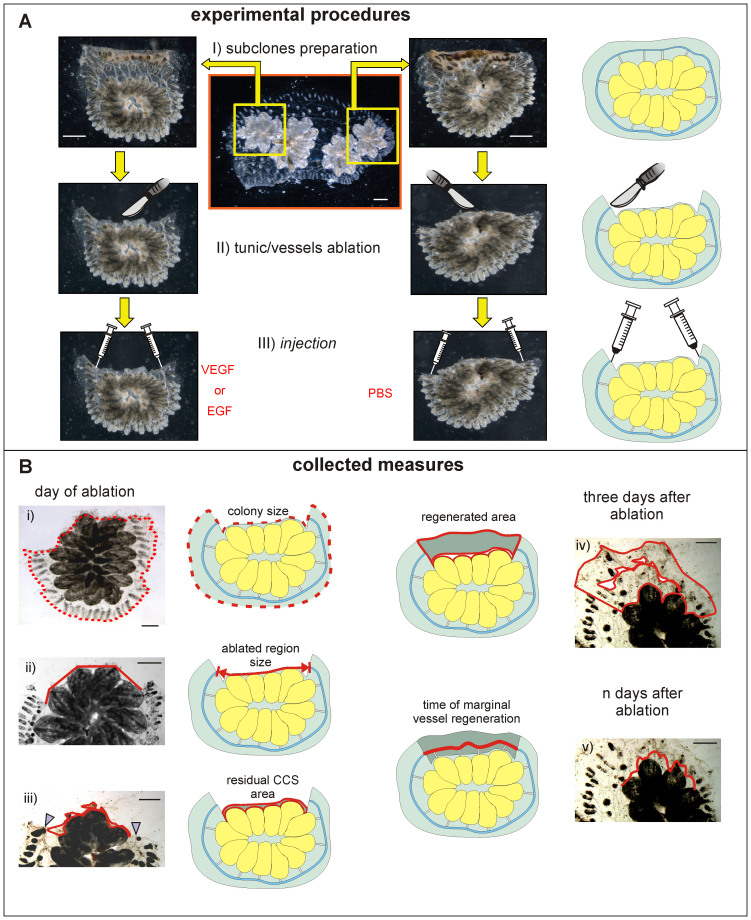
Experimental procedure and collected measures. (A) Scheme of the three steps performed in this work for the injection of pairs of ablated clones. I) subclone preparation (from fragments of the same parental colony); II) tunic and vessels ablation in front of four to five zooids); III) parallel injection (one with the tested growth factor, the other with the solvent). (B) View of the five measures collected for each subclone (see Supplementary Table S1 for detailed information). Three measurements were collected the day of ablation, a few hours after the operation: i) colony size (dotted line); ii) ablated region size, calculated as the distance between lateral cut edges (polyline); iii) residual CCS area, delimited from the curved line in the ablated region. Two measurements were collected the following days: iv) the regenerated area after three days, delimited from the curved lines, which inner one delimitates the subtracted residual postoperative CCS area); v) days required for the regeneration of the marginal vessel (identified, in the ablated region, from the curved line). Arrowheads, marginal vessel stumps. Each step for the experimental procedure in (A) and for the measurements in (B) is flanked by a sketch. Scale bars: 1 mm. Images were organized with CorelDRAW X4 (Corel Corporation). Sketches were prepared with CorelDRAW X4 (Corel Corporation).

Colonies were observed daily to follow the complete regeneration of the marginal vessel (Supplementary Table S1, Fig. 2 D–F, Fig. 3 B). They were examined thoroughly three days after injection and photographed to measure the areas of interest (Supplementary Table S1, Fig. 3 B). Regeneration was recognisable in all injected colonies from 1 day after ablation onward: a thin line of new tunic was penetrated by growing vessel stumps, and new vessels and small ampullae bordered the cut edges. In the following days, regeneration proceeded, and the tunic re-covered the ablated area to reach the usual external profile. The stumps of marginal vessels were involved in the regeneration of vessels, which progressively colonised the new tunic. They came into contact and fused with nearby regenerating stumps of radial vessels. New vessels were often produced by bifurcation and sprouts from their walls. After a few days, the marginal vessel was fully regenerated, and the network of vessels was restored in the ablation area (Fig. 2 C–F).

**Figure 4:**
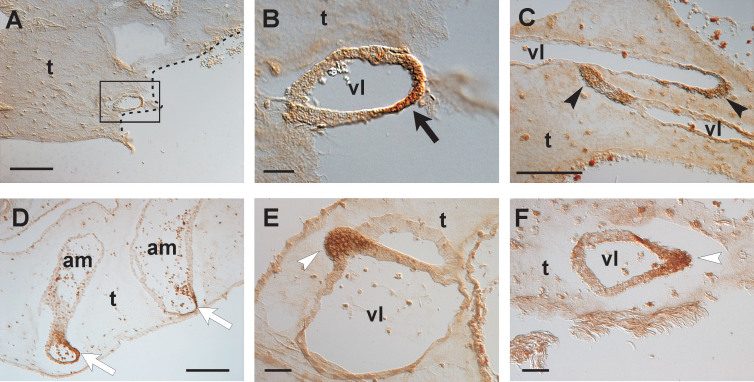
Immunostainig with antibodies against vertebrate angiogenic factor receptors. Selected sections representative of *B. schlosseri* regenerating CCS in colonies injected with PBS. (A–C) The antibody antigen is EGFR. In (A) the dotted line indicates the cut edge of an operated colony two hours after the injection of PBS (approximately four hours after ablation of the CCS); (B) represents the boxed area in (A); the section in (C) is referred to an operated colony three days after ablation. (D, E) The antibody antigen is VEGFR-1; regenerating regions of operated colonies three days after ablation. (F) The antibody antigen is VEGFR-2; regenerating region of an operated colonies one day after ablation. Black arrowheads, stained apexes of elongating vessels (vl); black arrow, stained vessel stump after ablation; white arrows, stained apexes of ampullae (am); white arrowheads, stained sprouts. t, tunic Scale bars 150 μm in (A, C and D); 50 μm in (B, E and F); 100 μm in (C).

Some control colonies (operated to remove the tunic and the CCS, and then injected with PBS) were also used to verify the localization of antibodies against VEGF and EGF receptors, *i.e.* VEGFR-1, VEGFR-2, and EGFR (Fig. 4). The antibodies specifically marked the CCS in regenerating regions: the stain was identifiable in vessel stumps, apexes of new ampullae and new sprouting vessels following excision. A slight background is at the tunic level. Also some tunic and blood cells result labeled.

### Statistical analyses: impact of VEGF and EGF in angiogenesis

The injected regenerating colonies were examined for the regenerated areas and days to regenerate the marginal vessel. Statistical analyses were performed to compare regeneration in colonies injected with a human growth factor *versus* colonies injected only with PBS. The statistical procedure is reassumed in Supplementary Table S2.

#### Dataset

A dataset was created (Supplementary Data S3) containing data regarding i) the “colony size” (a couple of hours after operation), ii) the “regenerated area” (3 days after ablation), iii) the “ablated region size” (distance between the lateral cut edges) and iv) the “time of marginal vessel regeneration” (days required to regenerate the marginal vessel) (Fig. 3 B and Supplementary Table S1).

#### Putative outliers identification

Preliminary analyses were conducted by the Grubbs test to identify putative outliers in each sample. For each test with a p-value ≤ 0.05, a data point with the highest value was considered to be an outlier. This point and its paired samples were removed. Then, subsequent tests were performed on both samples until p-values > 0.05 were reached simultaneously (the test procedure can be found in the Supplementary Data S4). Each putative outlier was further controlled checking its healthy parameters. Reports and set of photos obtained during daily observations after injection always confirmed their healthy condition. Moreover, measures related to the putative outliers were checked to avoid any error in measurements. No putative outliers were related to any error in evaluation of a healthy condition and/or measurements. As a consequence, the putative outliers were maintained in the dataset.

#### Normality and homogeneity of variance tests

A second preliminary statistical test procedure was performed to determine if each sample was normally distributed (Shapiro test procedure is in Supplementary Data S5). For a p-value > 0.05 the null hypothesis of normality was accepted. The following samples resulted in p-values ≤ 0.05: the VEGF-injected sample related to regenerated areas, the PBS-injected samples related to the colony sizes, the PBS-injected samples related to the regenerated areas, and all the samples related to the time of marginal vessel regeneration (both injected with PBS and with growth factors). A base-10 logarithmic (log_10_) transformation was consequently performed on the related paired samples (transformed samples are in Supplementary Data S3). Some of the transformed samples maintained a non-normal distribution with a p-value ≤ 0.05: i) the control (PBS-injected) sample paired to EGF-injected sample with regards to the regenerated areas and ii) all the samples related to the time of marginal vessel regeneration (see Supplementary Data S5).

A third procedure, the Levene test, was then performed to test the hypothesis of homogeneity of variances between each normally distributed pair of samples (see Supplementary Data S6). The test output indicated homogeneity of variances between the pairs of samples related to the starting conditions (*i.e.*, ablated region size and the log_10_ transformed samples related to the colony size).

As a consequence, parametric statistical methods were conducted on samples related to the starting conditions but performed using the log_10_ transformed data for the colony size-related samples. Conversely, samples related to the regenerated area and the time of marginal vessel regeneration were further analysed by means of non-parametric methods.

#### Checking the homogeneity of paired samples at the starting conditions: treated and control subclones form homogeneous samples with regards to dimension and ablated region size

**Figure 5:**
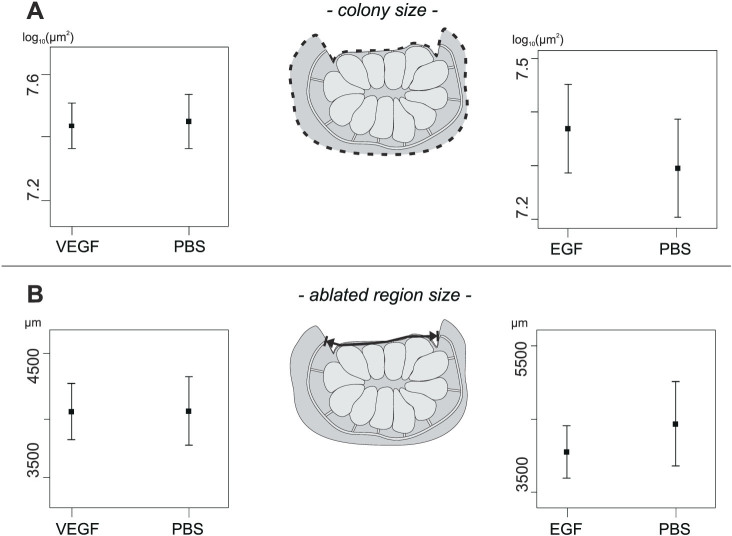
Plots showing that the intervals of the means (0.95% confidence limits) of each paired samples at starting conditions largely overlap. (A) Intervals of the means related to the size of the colonies injected with a growth factor (VEGF or EGF) and of the respective controls (injected with PBS). (B) Intervals of the means related to the ablated region sizes of colonies injected with a growth factor (VEGF or EGF) and of the respective controls (injected with PBS). Each measurement related to paired samples in A and B is sketched similarly to Fig. 3.

At this point, the homogeneity of each paired sample sets at the starting conditions (log_10_ of colony sizes and ablated region sizes) was checked and the hypothesis accepted by means of the confidence intervals of the means (Fig. 5, procedure to obtain plots is in Supplementary Data S7). This permitted the assertion that the following statistical analyses (about regeneration between colonies injected with a growth factor and with PBS) were not influenced by any diversity in colony size and ablated region size between the two samples.

#### Correlations between regeneration and starting conditions: chosen sets of colonies regenerate CCS independently from their dimension and ablated region size

**Figure 6:**
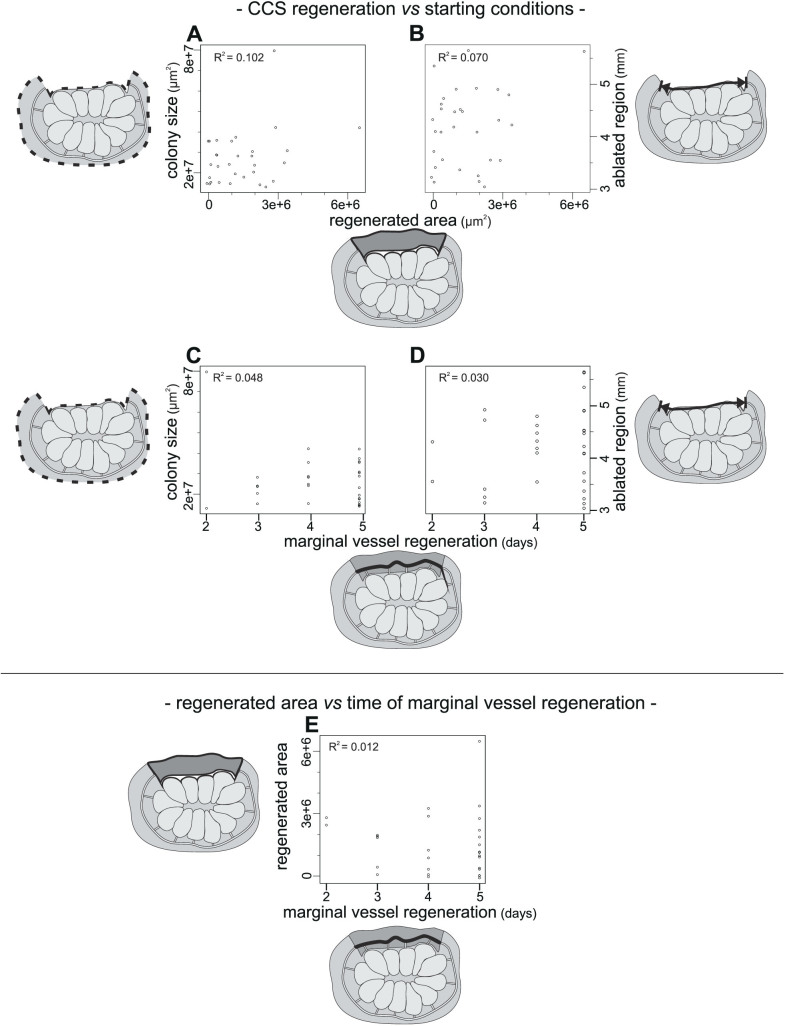
Plots of the linear regression analyses and respective R^2^. (A–D) Regression plots showing the relationship between a regeneration parameter (regenerated area or time of marginal vessel regeneration) and a starting condition parameter (colony size or ablated region size) in the colonies injected with PBS. (E) Regression plot showing the relationship between the two regeneration parameters in the colonies injected with PBS. Each plot is flanked by the sketches of the analysed parameters.

The following step was performed to determine if any correlation existed between CCS regeneration (with regards to both the regenerated area and the time of marginal vessel regeneration) and the starting conditions (colony size and ablated region size). For this step, all the data from colonies injected with PBS were used. Conversely, data from colonies injected with grow factors were excluded from the analysis in order to avoid the introduction of the variable of their impact in the correlation test. The four new samples (described in Supplementary Table S8) containing data of colony sizes, ablated region sizes, regenerated areas and time of marginal vessel regeneration, respectively, were each composed of 31 data points (dataset in Supplementary Data S9). Both the resulting plots of the linear regression and respective coefficient of determination (R^2^) (Fig. 6 A–D, procedures are in Supplementary Data S10) always indicated an absence of correlation. This meant that the chosen set of colonies regenerated the CCS with respect to both the regenerated area and the time of marginal vessel regeneration, independently from their whole dimension and the size of the ablated region.

#### Correlations between regeneration parameters: time of marginal vessel regeneration is independent from tunic regeneration

A similar correlation analysis was also conducted between the two samples containing data on the regenerated area and the time of marginal vessel regeneration, each formed by the respective observations from colonies injected with PBS. Additionally, the resulting plot of the linear regression and respective R^2^ (Fig. 6 E, see Supplementary Data S10 for test procedure) indicated an absence of correlation, indicating that the two variables were independent of each other.

#### Testing the impact of the injected factors on CCS regeneration: VEGF and EGF promote angiogenesis

All of the above tests allowed the analysis of the means of paired samples related to CCS regeneration to test if they were equal. They also induced selection of the non-parametric Wilcoxon signed-rank test for both the samples related to regenerated areas and the samples related to the time of marginal vessel regeneration. Log_10_ transformed data of regenerated areas were used for the VEGF-related paired samples because they resulted in a normal distribution, which was different from the other paired samples (see Supplementary Data S11).

**Figure 7:**
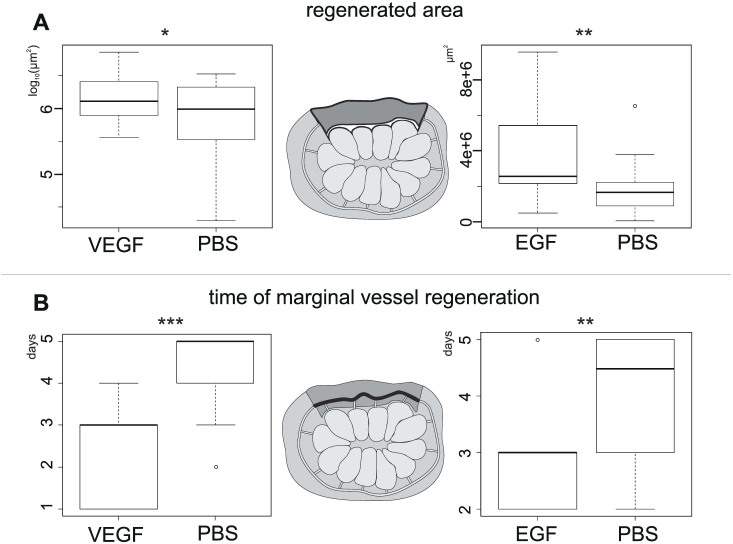
Boxplots showing data distributions between paired samples related to regeneration parameters. (A) Regenerated area of the colonies injected with a growth factor (VEGF or EGF) and of the respective controls (injected with PBS). (B) Time of marginal vessel regeneration of the colonies injected with a growth factor (VEGF or EGF) and of the respective controls (injected with PBS). Tests of equality of the means (Wilcoxon signed-rank test), resulting p-values: * < 0.05; ** < 0.01; *** < 0.001. Each measurement related to paired samples in A and B is sketched.

The boxplots in Fig. 7 show that: i) the medians of the samples related to regenerated areas of the colonies injected with both human VEGF and EGF are higher with respect to their controls (paired t test p-values: 0.013 and 0.003, respectively); ii) the medians of the samples related to the time of marginal vessel regeneration of the colonies injected with both human VEGF and EGF are lower with respect to their controls (Wilcoxon signed-rank t test p-values: 5e-4 and 0.009, respectively). The results significantly support the hypothesis that both human VEGF and EGF induce a higher regeneration in *B. schlosseri* CCS in terms of i) production of tunic and embedded vessels; and ii) velocity to fully reform the marginal vessel.

## Discussion

In this study, we propose the colonial circulatory system of *Botryllus schlosseri* as a model for *in vivo* angiogenic studies, introducing a methodology to quantitatively analyse the effect of an injected solute. Our analysis is strictly associated with the peculiarity of being able to easily obtain pairs of subclones, each formed from pieces of the same colony, essentially constituting biological replicas. Subclones are identical genetically and functionally and are all at the same developmental stage^20^. This is an important added value because measured parameters between subclones can be evaluated considering that they are not perturbed by intrinsic variables, so that differences derive only from external perturbations (*e.g.*, experimental variations). This feature allows the use of the respective collected data for paired sample analysis because each data point in one of the paired samples (control or treated with the angiogenic factor) was matched to a unique data point in the second sample.

Similarly, isogenic strains of several organism, including mammals, are also appropriate paired biological samples. Isogenic strains are formed by genetically identical individuals obtained by inbreeding for several consecutive sexual generations. Their higher genetic uniformity with respect to outbred stocks leads to more sharp experiments and strongly facilitate the achievement of a given level of statistical power^33^ result invaluable also for biomedical issues related to angiogenesis^34^,^35^. Nevertheless, *B. schlosseri* subclones, as pieces of the same colony propagated asexually, are not affected by problems deriving by genetic drift and by the cryptic genetic variations existing among the individuals of isogenic strains^36^. Moreover, as animal model, *B. schlosseri* offers the advantages of: absence of ethical constrains, facility to rear, cheapness to maintain in lab, and a CCS growing fast which is directly accessible and visible under the stereomicroscope.

It should be noted that for *in vitro* studies, clonal propagation is typical and simple to obtain from cell cultures, as opposed to experimental animal models. In chordates, only colonial tunicates undergo both sexual and asexual reproduction^37^. Within the tunicate species, a defined protocol of rearing has been established for *B. schlosseri*^21^ and is at present used in different laboratories as well as our own^28^,^38^,^39^. This confirms the utility of *B. schlosseri* as a model species^19^ for experimental studies, especially considering that its annotated genome has recently become available^40^.

The preliminary study design, composed of several steps (outlier identification, normality and homogeneity of variances tests, homogeneity of paired samples at starting conditions, and correlations between regeneration and starting conditions) allowed for control and optimisation of the datasets to better determine if the injected protein affected CCS angiogenesis. In particular, the use of pre-test measurements (colony size and ablated region) produced a more powerful test of equality of the means between the paired samples (*i.e.*, between colonies injected with a grow factor and with PBS) with respect to a study design with no pre-test data. Indeed, we demonstrated that the difference in both the dimension of the chosen set of colonies and the related performed operations, with regards to the size of the ablation, did not interfere with the following statistical analyses.

As a parameter to quantify angiogenesis, we used the area of regenerated tunic. The choice of this indirect parameter was based on previous results demonstrating i) a strong correlation, in that more tunic production corresponds to more vessel formation; and ii) that angiogenesis similarly occurs during CCS regeneration and normal *B. schlosseri* colony propagation, with the advantage that regeneration occurs in an experimentally delimited area (the ablated region), which is easy to analyse^13^. Moreover, even if the whole quantification of vessel formation in the ablated region is feasible, the measurement of regenerated tunic is simpler and less subject to errors.

We also evaluated angiogenesis by another parameter: the time of marginal vessel regeneration that is directly related to this process. Previous results^13^ showed that the marginal vessel stumps are indeed involved in the angiogenic process. They elongate and progressively colonise the new tunic, often coming into contact and fusing with nearby growing vessels (both new vessels and the residues of radial ones). Eventually, the marginal vessel is fully reformed, re-establishing the continuity of the related blood circulation. Also in this case, we had to consider the meaning of the demonstrated requisites of homogeneity of paired samples with regards to the ablated region size and the homogeneity of variances between them. The test of equality of the means between treated and control subclones with regards to the time of marginal vessel regeneration, required that the marginal vessels and the other involved stumps were equally involved between the two samples. This having been reached verified that the homogeneity of variances and controlling of the distance between the marginal vessel stumps were homogenous. Moreover, because each zooid develops only one radial vessel, the constant number of zooids affected by ablation resulted in a similar quantity of involved radial vessel stumps.

The linear regression analysis between the two variables related to regeneration (the regenerated tunic area and the time of marginal vessel regeneration) showed that there is no correlation, indicating an independence between them. The reason for this independence can be found by analysing the morphodynamic process underlying the CCS regeneration. In the regenerating region, a few elongating sprouting stumps and some new sprouts are involved in marginal vessel regeneration, whereas both ampullae and sprout epithelia are involved in tunic formation^13^. Once vessel stumps have sufficient tunic matrix to grow and reform the marginal vessel (this occurs in the tunic proximal to the zooids), this process may proceed independently from the formation of new tunic in the remaining ablated area.

The aforementioned independence permitted an evaluation as to whether the human VEGF and EGF treatment affected both the tunic production and vessel elongation.

The initial concentration of both VEGF and EGF that was injected showed that they significantly induced angiogenesis. No other concentrations was used to test their impact on *B. schlosseri*. This because our goal was not to detail the role of VEGF and EGF in the CCS, but to individuate and detail an useful experimental protocol and data analysis procedure which permits to determine if an injected molecule significantly induces angiogenesis from a morphodynamic point of view. For this, we used two pro angiogenic human factors at a specific dose and concentration. In both cases the stringent statistical analyses of collected measures resulted positive. We cannot exclude the possibility that different amounts of them may result in a stronger and/or in a toxic effect. Further experiments will be necessary to verify it, which eventually results in a dose-response curve.

From an evolutionary point of view, and for a perspective evaluation of the vessel network model, it has to be noted that in vertebrates the circulatory system is closed, and the blood flows into vessels delimited by endothelial cells. Conversely, the vascular system of invertebrates is commonly composed of cavities (sinuses and lacunae) not delimited by an epithelium (open circulatory system). Therefore, blood flows among organs, contacting them directly at their basement membrane level. This invertebrate haemal system organisation has also been demonstrated in tunicates, animals that are evolutionarily the closest to vertebrates^14^,^41^. Therefore, there are important structural differences between invertebrate and vertebrate circulatory systems. The differences include their embryonic origin, being always mesodermic in vertebrate vessels, but is different in invertebrates, where it is commonly not delimited by the endothelium. However, *B. schlosseri* couples to a typical invertebrate intrazooidic open circulatory system, which occupies zooid cavities, an interzooidic vascular system (*i.e.*, the CCS). The CCS is composed of blood vessels delimited by the basement membrane of a simple epithelium of epidermal origin (therefore ectodermal) that extends outside the zooids into the tunic. Comparison between the *B. schlosseri* CCS and the vertebrate vasculature shows that, even if the organisation and embryonic origin are not the same, vessel formation is similar at a morphodynamic level, because both use a typical angiogenic sprouting mechanism. Moreover, immunohistochemical analyses indicate that *B. schlosseri* angiogenic pathways correspond to those of vertebrates: antibodies against human EGF, FGF2, VEGF and their receptors specifically recognise angiogenic sites^13^. The immunohistochemical experiments here performed on control colonies (regenerating colonies injected with PBS) clearly evidenced that antibodies against vertebrate VEGF and EGF receptors localized in vessel stumps and sprouts of the regenerating area. This antibody localization supports the hypothesis that the two factors play a role in *B. schlosseri* angiogenesis. The molecular pathway under the control of VEGF, the main angiogenic factor in vertebrates, has been investigated in *B. schlosseri* at the molecular level^25^,^26^. The orthologous ligand has been characterised and functionally tested, demonstrating that its repression negatively influences vessels formation^25^.

It is noteworthy that blood spaces that are not lined by an endothelium can also be found in vertebrates^42^. This organisation is adopted by some tumour cells, due to a *de novo* vessel formation mechanism referred to as vasculogenic mimicry^43^. The structure of tumour-associated channels formed by vasculogenic mimicry differs from typical vertebrate blood vessels in that there is no endothelial cell lining. Although the exact function of a vasculogenic mimicry network remains to be elucidated, it mimics the function of normal vessels, distributing plasma and blood to the tissue^43^. Moreover, gene expression analysis demonstrated that tumours capable of vasculogenic mimicry upregulate genes involved in angiogenesis and vasculogenesis (reviewed in^44^), and the pathways related to VEGFs, FGF2, EGF and/or their receptors seem to play key roles during this mechanism of vessel formation^45^,^46^,^47^. All of these findings, as Kucera and Lammert recently noted^42^, open new perspectives in studying the role of angiogenesis/vasculogenic mimicry in tumour growth and motivate investigation of the molecular mechanisms of invertebrate vascular tube formation, which may reveal novel and useful targets for anticancer therapy.

In conclusion, these and previous results encourage other preliminary studies that may help to elucidate whether *B. schlosseri* CCS could be an effective complementary model for *in vivo* studies related to circulation and anticancer therapy. More generally, the characteristics of the *B. schlosseri* CCS evidenced in this and previous studies^13^,^14^,^25^,^26^, in addition to the key phylogenetic position of tunicates as sister group of the vertebrates^48^, indicate that the *B. schlosseri* CCS can be considered a useful and highly accessible experimental model for *in vivo* angiogenic studies. Here we have demonstrated that the pro-angiogenic human EGF and VEGF^29^,^30^, which are known to activate crucial signalling cascades for the growth of several human tumours^49^,^50^,^51^, similarly induce angiogenesis in *B. schlosseri* CCS. This evidences its potentiality to contribute to the understanding of angiogenic mechanisms and their evolution in chordates. In this respect, it may be of interest to evaluate other levels of functional similarity of VEGF and EGF between humans and *B. schlosseri*. For example, in recently published pilot observations of colonies injected with a tyrosine kinase inhibitor, there was a preferential affinity for the VEGFRs that appeared to block CCS regeneration^25^. These studies attempt to provide and encourage an in depth analysis of the unusual vasculature of *B. schlosseri*, taking into consideration the origin, nature, and roles of the cellular and molecular agents of its growth^26^, thus, as warranted by Muñoz-Chápuli^16^, avoiding the risk of an oversimplification of the mechanisms involved in angiogenesis.

## Methods

### Animal rearing and *in vivo* techniques

Following Sabbadin's technique^21^ (reviewed in^19^), colonies of *B. schlosseri*, collected from the Lagoon of Venice, were adhered to glass slides and cultured in seawater in the laboratory at 18°C.

Pairs of subclones prepared by fragmenting a same-parental colony were positioned on glass slides and left to adhere for 1–2 weeks (Fig. 3 A). Then, the surgical procedure was performed to induce CCS regeneration as previously described^13^.

After two to four hours, both replicas for each pair derived from the same parental colony were injected in parallel with the protein of interest dissolved in the appropriate physiological solution; injection of the diluent only served as a control. Vessels and/or ampullae close to the ablated area were punctured using thin glass micropipettes prepared with a Narishige PD-5 horizontal capillary puller; the injection system consisted of a PLI-100 Pico-Injector (Medical Systems Corp.), a Singer Mk1 micromanipulator and a Leica MZ6 Stereomicroscope.

The proteins injected in this study were recombinant human EGF (Peprotech, cat n. 100-15) and recombinant human VEGF_165_ (Peprotech, cat n. 100-20), both eluted in PBS.

Colonies were observed using a Leica MZ16F stereomicroscope. They were photographed with a Leica DFC480 digital camera controlled by Leica IM500 Image Manager; the same software was used to measure the areas of interest in μm^2^. Images were organized with CorelDRAW X4 (Corel Corporation). Sketches were prepared with CorelDRAW X4 (Corel Corporation).

### Immunohistology

We analyzed the responses to anti-VEGFR-1, anti-VEGFR-2, and anti-EGFR (Santa Cruz Biotechnology; cat numbers sc-9029, sc-504 and sc-03, respectively) in three control colonies (operated and then injected with PBS) following a procedure previously described for an immunoperoxidase protocol^13^,^14^. Following this procedure also the negative (no primary antibody) and the positive (with anti-sea urchin α-tubulin antibody, Sigma-Aldrich, cat. n. T-5168) controls was carried out for each experiment. These gave the expected results, since the sections showed no stain (negative controls) and brown-stained ciliary/cytoplasmic microtubules (positive controls). The three colonies utilized for the immunohistology were fixed at different times after ablation (four hours, one day and three days, respectively).

### Statistical analyses

Statistical analyses were performed using R Software Environment, version 3.0.1^52^. The aim of the analyses was to determine if the means of the two samples were equal with tests for paired data (pairs of subclones injected in parallel with the protein of interest and PBS). If a data point was eliminated, the paired data point was also removed. The Grubbs test (*grubbs.test* function of *outliers* package^53^) was used to detect the presence of outliers. Confidence intervals of the means, with a confidence limit of 95%, were plotted using the *error.bars.by* function of *psych* package^54^ to check the homogeneity of samples at the starting conditions with regard to colony size and the ablated region size. The Shapiro test on *stats* package (*shapiro.test* function)^52^ was used to determine if each data set was normally distributed, and the Levene test (*leveneTest* function in *car* package)^55^ was used to test the homogeneity of variances between each sample injected with the protein of interest and the respective control, both for regenerated CCS areas and starting condition samples. If necessary, the log_10_ transformation was applied to try to reach normality of a sample and homogeneity of variances between samples^56^. Box-and-whisker plots were produced with *boxplot* function (*graphics* package^52^).

The non-parametric Wilcoxon signed-rank or the parametric paired t test (*via* either *t.test* or w*ilcox.test* function of *stats* package^52^) was subsequently chosen to test if the means of the two samples were equal. The Wilcoxon signed-rank test for paired data was first conducted on samples at starting conditions to verify their homogeneity with regards to colony sizes and ablated region sizes. This test was performed instead of the confidence intervals of the means if at least one sample of the pair resulted not normally distributed and/or the hypothesis of homogeneity of variances between a normally distributed pair of samples was rejected. The Wilcoxon signed-rank or the paired t test were then conducted on samples related to regenerated CCS (both the number of days to regenerate the marginal vessel and the regenerated area after three days) to investigate if the injected factor affected CCS regeneration.

The coefficient of determination (R^2^) of a linear regression was performed (*via* *R2.lm* function of *REGRDIAGNOSTICS* tool in *nsRFA* package^57^) to determine correlations between the regeneration and starting conditions (*i.e.*, if either the regenerated CCS area or the number of days to regenerate the marginal vessel correlates with the colony size or ablated region size) and between the regenerated area and the marginal vessel regeneration. Linear regressions were fitted by *lm* function of *stats* package^52^, regression plots were produced with the *plot* function of the *graphics* package^52^. Plots were organized with CorelDRAW X4 (Corel Corporation).

## Author Contributions

F.G. conceived the study, performed the statistical analyses, drafted the manuscript, prepared figures, participated in the animal rearing, study design and its coordination and helped to collect and rear animals and to perform the experiments. F.C. participated in the animal collection, experiments execution and study coordination, and helped to draft the manuscript. F.R. participated in the animal rearing, experiments execution, study design and helped to draft the manuscript. G.Z. participated in the animal rearing, experiments execution, study design, its coordination, helped to conceive the study and to draft the manuscript. L.M. participated in the animal collection, study design and its coordination, helped to conceive the study, to perform the statistical analyses, to draft the manuscript and to prepare figures. All authors read and approved the final manuscript.

## Supplementary Material

Supplementary Information Supplementary information

## Figures and Tables

**Figure 1:**
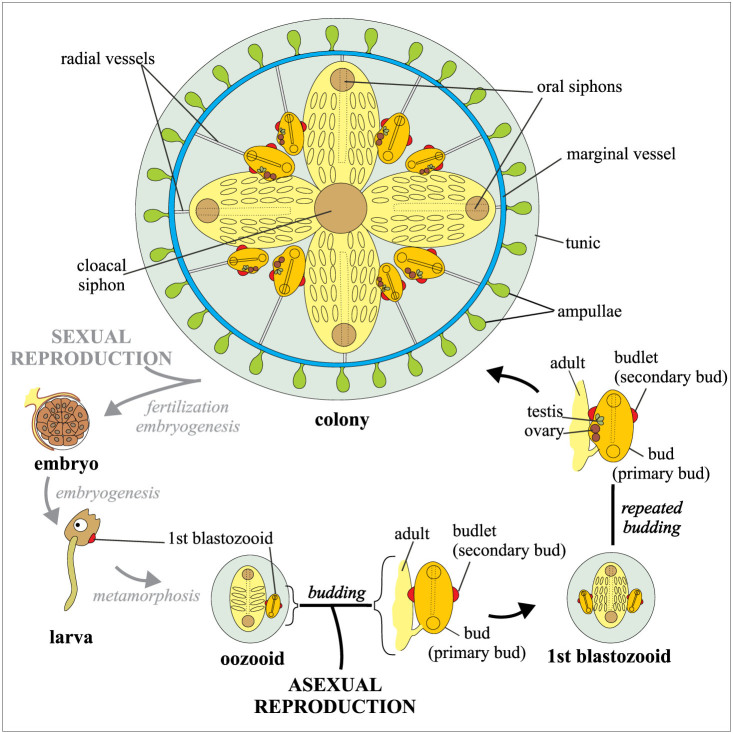
*Botryllus schlosseri* life cycle. Ozooid and blastozooids are shown in a dorsal view. A colony is represented as formed by a single system of four adult blastozooids each bearing two buds, that in turn bear two budlets; modified from^20^. Sketches were prepared with CorelDRAW X4 (Corel Corporation).
