# Supplementary material for: Testing an unusual in vivo vessel network model: a method to study angiogenesis in the colonial tunicate Botryllus schlosseri
Source: Sci Rep. 2014 Sep 24;4:6460. doi: 10.1038/srep06460 (PMC4173039; doi:10.1038/srep06460)
Supplement: Supplementary Information [file srep06460-s1.pdf]

MANUSCRIPT TITLE: Testing an unusual *in vivo* vessel network model: a method to study angiogenesis in the colonial tunicate *Botryllus schlosseri*

AUTHORS: Fabio Gasparini, Federico Caicci, Francesca Rigon, Giovanna Zaniolo, Lucia Manni

Supplementary information – Tables describing datasets and test procedures. Datasets and test procedures performed in R (Command lines in red).

Table S1. **Description of collected measures.** Table describing the collected measures sketched in Fig. 3 B, and which are in Supplementary Data S3.

Table S2. **Schematized description of performed data analysis procedure.** Table schematizing the performed data analysis procedure.

Data S3. **Dataset of collected measures.** Dataset of collected measures to import into R. Base-10-log transformed paired samples of the pair resulted not normally distributed are inserted.

Data S4. **Procedure for outliers identification.** Test procedure performed in R for outliers identification starting from dataset in Supplementary Data S3.

Data S5. **Procedure for testing normality distribution of samples.** Test procedure performed in R for testing normality distribution of samples starting from dataset in Supplementary Data S3.

Data S6. **Procedure for testing homogeneity of variance across normally distributed paired samples.** Levene's test performed in R for homogeneity of variance across normally distributed paired samples.

Data S7. **Procedure to plot intervals of the means.** Procedure used in R to plot intervals of the means (0.95% confidence limits) of starting conditions for paired samples.

Table S8. **Description of samples used for correlation analyses.** Table describing the samples used for correlation analyses, which are in Supplementary Data S9.

Data S9. **Dataset of aggregated control samples.** Dataset to import the aggregated control samples in R and to obtain, following the instruction in the Supplementary Data S10, the regression plots and the coefficients of determination (R^2) of the regression.

Data S10. **Procedure to obtain the regression plots and the R^2.** Procedure performed in R to obtain the regression plots and the coefficients of determination (R^2) of the regression, starting from dataset in Supplementary Data S9.

Data S11. **Procedures to obtain the boxplots and to test if the means of the paired samples are equal.** Procedures performed in R to obtain the boxplots of the paired samples related to the regeneration of the CCS available in Supplementary Data S3, and to test if the means of the same paired samples are equal.

SUPPLEMENTARY TABLE S1.

Description of collected measures sketched in Fig. 3 and which are in Supplementary Data S3. *: log_{10} transformed samples

COLLECTED MEASURES	DESCRIPTION OF SAMPLE TYPES USED FOR STATISTICAL ANALYSIS	names of VEGF and EGF related samples (ctrl: control)	NOTES
Colony sizes after operation (μm^2)	samples at starting conditions in term of colony size	AreaTot_d0_VEGF<br>AreaTot_d0_VEGFctrl<br>AreaTot_d0_EGF<br>AreaTot_d0_EGFctrl	Area occupied by the whole specimen after the procedure of CCS ablation in front of four to five zooids
Ablated region sizes (μm)	samples at starting conditions in term of region involved in ablation	DistLCedges_d0_VEGF<br>DistLCedges_d0_VEGFctrl<br>DistLCedges_d0_EGF<br>DistLCedges_d0_EGFctrl	Distance between the lateral cut edges calculated, the day of ablation, passing a polyline between them and tangent to zooids involved in the ablation
Residual CCS areas (μm^2)			Area composed by the residues of tunic and CCS remained after the ablation between and in front of the involved zooids
Regenerated areas after 3 days (μm^2)	regenerated CCS area of samples	AreaReg_d3_VEGF<br>log10_AreaReg_d3_VEGF*<br>AreaReg_d3_VEGFctrl<br>log10_AreaReg_d3_VEGFctrl*<br>AreaReg_d3_EGF<br>AreaReg_d3_EGFctrl	CCS area between and in front of zooids involved in the experimental procedure, measured three days after the operation (after subtraction of the residual postoperative CCS area)
times of marginal vessel regeneration (days)	samples that describe how long the marginal vessels have required to fully regenerate	DaysMVregVEGF<br>DaysMVregVEGFctrl<br>DaysMVregEGF<br>DaysMVregEGFctrl	Days required to regenerate the marginal vessel from its stumps in lateral cut edges and radial vessel stumps in proximal cut edge

SUPPLEMENTARY TABLE S2

Schematized description of performed data analysis procedure.

TEST/procedure	RATIONALE	ANALYSED SAMPLES	NOTES
**1) Grubbs test**	to identify putative outliers	all	Analysis of resulting putative outliers to avoid error in healthy evaluation and/or measurements
**2) Shapiro test**	to test if dataset is normally distributed	all	Test performed after $\log_{10}$ transformation of non normally distributed paired samples
**3) Levene test**	to test homogeneity of variances between normally distributed paired samples	normally distributed paired samples	Test performed on normally distributed $\log_{10}$ transformed paired samples.
**4) $\mathbf{Log_{10}}$ transformation**	to attempt to reach normality of a sample or variance homogeneity between paired samples	samples not normally distributed or with absence of variance homogeneity in the pair	
**5) Confidence intervals of the means**	to check homogeneity of paired samples	starting condition related paired samples	performed in case of paired samples both normally distributed and homogeneous in term of variance
**6) Coefficient of determination (R^2) of a linear regression**	to determine if correlations exist between collected measures	(i) all samples containing data from colonies injected with PBS (see Table 3)<br>***(ii) samples related to regeneration containing data from colonies injected with PBS*** (see Table 3)	(i) to verify if the set of colonies we used regenerated independently from their starting conditions<br>***(ii) to investigate if the two samples containing the respective information on regeneration correlates***
**7) Wilcoxon signed-rank** (non-parametric) or **Paired t** (parametric) **test**	to test if the means of paired samples are equal	(i) paired samples related to starting condition<br>***(ii) paired samples related to regeneration***	(i) Wilcoxon signed-rank test performed instead of the confidence intervals of the means (see point 5) to verify the homogeneity at starting conditions<br>***(ii) to investigate if the injected molecule impacted on CCS regeneration***

SUPPLEMENTARY DATA S3

DATASET OF COLLECTED MEASURES TO IMPORT INTO R
(base-10-log transformed paired samples when one
of the pair resulted not normally distributed).

```
# colony size after operation

# VEGF

AreaTot_d0_VEGF <- scan()
38781059 27135498 41183285 30694319 25149833
23847573 36194395 32534563 23329538 22300727
12616061 19777362 20336433 28608767 18963624
44107230 22121297 27808679 53333178

log10_AreaTot_d0_VEGF <- scan()
7.588619664 7.433537796 7.614720985 7.487058002 7.400535106
7.377444187 7.558641322 7.512344978 7.367906138 7.348319021
7.100923780 7.296168363 7.308274780 7.456499141 7.277921336
7.644509784 7.344810587 7.444180359 7.726997463

AreaTot_d0_VEGFctrl <- scan()
35531054 37505911 35566298 14886998 25011034
15476291 30866356 23251402 17772963 36006383
28329622 35527648 24131725 14194599 42262730
79812606 28833941 30521187 28737823

log10_AreaTot_d0_VEGFctrl <- scan()
7.550608091 7.574099719 7.551038663 7.172807130 7.398131647
7.189666887 7.489485361 7.366449145 7.249759837 7.556379497
7.452240780 7.550566457 7.382588368 7.152123128 7.625957547
7.902071491 7.459904005 7.484601420 7.458453866

# EGF
AreaTot_d0_EGF <- scan()
20461702 16755229 36108996 39424820 22708038
13307111 22906696 23258796 21325848 25587103
20866201 30005176
```

```
      log10_AreaTot_d0_EGF <- scan()
      7.310941755 7.224150368 7.557615413 7.595769719 7.356179612
      7.124083780 7.359962452 7.366587230 7.328906309 7.408021117
      7.319443387 7.477196179

AreaTot_d0_EGFctrl <- scan()
14846368 17941039 15978138 13163135 23746737
14686625 15411596 24247940 19528538 42121133
28276128 20481610

      log10_AreaTot_d0_EGFctrl <- scan()
      7.171620221 7.253847590 7.203526168 7.119359335 7.375603942
      7.166922006 7.187847616 7.384674849 7.290669731 7.624500045
      7.451419939 7.311364092



                              # regenerated area after 3 days

                                    # VEGF
AreaReg_d3_VEGF <- scan()
1342797 791890 786721 1895263 2798093
692850 7328119 1684862 760879 1048446
366117 2512628 2139310 5622015 1724609
3424390 1100226 1586252 589120

      log10_AreaReg_d3_VEGF <- scan()
      6.128010362 5.898664859                         6.446862145
      5.840639221 6.864992513 6.226564335 5.881315598 6.020546067
      5.563619895 6.400128195 6.330273721 6.749892000 6.236690648
      6.534583220 6.041481904 6.200372183 5.770203767

AreaReg_d3_VEGFctrl <- scan()
19885 1167173 -22465 -82765 3258803
84775 3382075 390894 42390 342345
1253436 984490 84538 2211244 2886969
2817288 335158 1881259 340019

      log10_AreaReg_d3_VEGFctrl <- scan()
      4.298525595 6.067135233                         6.513058107
      4.928267798 6.529183234 5.592059004 4.627263417 5.534463989
      6.098102164 5.993211309 4.927051969 6.344636668 6.460442120
      6.449831245 5.525249590 6.274448591 5.531503186
```

```
                                  # EGF

AreaReg_d3_EGF <- scan()
3482386 1624747 9525507 6240232 2474587
497737 1994818 2548659 2280122 8193722
4624606 2573152

      log10_AreaReg_d3_EGF <- scan()
      6.541876908 6.210785744 6.978888101 6.795200736 6.393502727
      5.696999926 6.299903278 6.406311733 6.357958085 6.913481225
      6.665074738 6.410465441

AreaReg_d3_EGFctrl <- scan()
922096 1513287 2776660 2464037 1971225
38440 441013 888858 1142205 6499956
1853451 1931061

      log10_AreaReg_d3_EGFctrl <- scan()
      5.9647761381 6.1799213013 6.4435227040 6.3916472249 6.2947361984
      4.5847833790 5.6444513916 5.9488323856 6.0577440570 6.8129104168
      6.2679811090 6.2857959929


                            # ablated region (lateral cut edges distance)

                                  # VEGF

DistLCedges_d0_VEGF <- scan()
4193 3784 4015 5368 3881
4029 4737 4202 3310 3966
3868 3617 4339 4536 3910
3797 3683 3568 3809

DistLCedges_d0_VEGFctrl <- scan()
3723 4522 4328 3228 4802
4104 4228 3563 3136 4533
4480 4908 3413 3045 3550
4316 4093 4089 4624
```

```
                                  # EGF

DistLCedges_d0_EGF <- scan()
3473 4443 4761 4620 3586
4776 4336 4359 3154 3819
3920 3362

DistLCedges_d0_EGFctrl <- scan()
4474 5648 4900 3556 3146
5353 4731 4188 3372 5630
4925 3259

                             # regenerated marginal vessels (days)

                                  # VEGF

DaysMVregVEGF <- scan()
3 4 2 3 2
1 1 1 1 3
4 3 3 1 1
2 3 3 4

     log10_DaysMVregVEGF <- scan()
     0.477121255 0.602059991 0.301029996 0.477121255 0.301029996
     0           0           0           0           0.477121255
     0.602059991 0.477121255 0.477121255 0           0
     0.301029996 0.477121255 0.477121255 0.602059991

DaysMVregVEGFctrl <- scan()
5 5 4 5 4
4 5 5 5 5
4 5 3 5 4
2 5 5 4

     log10_DaysMVregVEGFctrl <- scan()
     0.698970004 0.698970004 0.602059991 0.698970004 0.602059991
     0.602059991 0.698970004 0.698970004 0.698970004 0.698970004
     0.602059991 0.698970004 0.477121255 0.698970004 0.602059991
     0.301029996 0.698970004 0.698970004 0.602059991
```

```
# EGF

DaysMVregEGF <- scan()
3 5 3 3 3
2 2 2 3 3
2 2

      log10_DaysMVregEGF <- scan()
      0.477121255 0.698970004 0.477121255 0.477121255 0.477121255
      0.301029996 0.301029996 0.301029996 0.477121255 0.477121255
      0.301029996 0.301029996

DaysMVregEGFctrl <- scan()
5 5 5 2 3
5 3 4 5 5
3 3

      log10_DaysMVregEGFctrl <- scan()
      0.698970004 0.698970004 0.698970004 0.301029996 0.477121255
      0.698970004 0.477121255 0.602059991 0.698970004 0.698970004
      0.477121255 0.477121255
```

```
# SUPPLEMENTARY DATA S4

# TEST PROCEDURE PERFORMED IN R FOR OUTLIERS IDENTIFICATION
## STARTING FROM DATASET IN SUPPLEMENTARY DATA S3
## (*): p-value < or = 0.05
## (in squared brackets are data identified as putative outliers)
library(outliers)

grubbs.test(AreaTot_d0_VEGF)            # p-value = 0.07833
grubbs.test(AreaTot_d0_VEGFctrl)        # p-value = 0.0001465 (*)   [79812606]
grubbs.test(AreaTot_d0_EGF)             # p-value = 0.1686
grubbs.test(AreaTot_d0_EGFctrl)         # p-value = 0.005771 (*)    [42121133]
grubbs.test(AreaReg_d3_VEGF)            # p-value = 0.005005 (*)    [7328119]
grubbs.test(AreaReg_d3_VEGFctrl)        # p-value = 0.5333
grubbs.test(AreaReg_d3_EGF)             # p-value = 0.1413
grubbs.test(AreaReg_d3_EGFctrl)         # p-value = 0.001164 (*)    [6499956]
grubbs.test(DistLCedges_d0_VEGF)        # p-value = 0.01014 (*)     [5368]
grubbs.test(DistLCedges_d0_VEGFctrl)    # p-value = 0.7226
grubbs.test(DistLCedges_d0_EGF)         # p-value = 0.612
grubbs.test(DistLCedges_d0_EGFctrl)     # p-value = 0.9108
grubbs.test(DaysMVregVEGF)              # p-value = 1
grubbs.test(DaysMVregVEGFctrl)          # p-value = 0.008078 (*)    [2]
grubbs.test(DaysMVregEGF)               # p-value = 0.006859 (*)    [5]
grubbs.test(DaysMVregEGFctrl)           # p-value = 0.3553


# then, after elimination of putative outliers and related data in paired samples:

grubbs.test(AreaTot_d0_VEGF)            # p-value = 0.0295 (*)      [53333178]
grubbs.test(AreaTot_d0_VEGFctrl)        # p-value = 0.7414
grubbs.test(AreaTot_d0_EGF)             # p-value = 0.1907
grubbs.test(AreaTot_d0_EGFctrl)         # p-value = 0.1794
grubbs.test(AreaReg_d3_VEGF)            # p-value = 0.002231 (*)    [5622015]
grubbs.test(AreaReg_d3_VEGFctrl)        # p-value = 0.3175
grubbs.test(AreaReg_d3_EGF)             # p-value = 0.01836 (*)     [9525507]
grubbs.test(AreaReg_d3_EGFctrl)         # p-value = 0.4267
grubbs.test(DistLCedges_d0_VEGF)        # p-value = 0.1462
grubbs.test(DistLCedges_d0_VEGFctrl)    # p-value = 0.4737
grubbs.test(DaysMVregVEGF)              # p-value = 1
grubbs.test(DaysMVregVEGFctrl)          # p-value = 0.04524 (*)     [3]
grubbs.test(DaysMVregEGF)               # p-value = 1
grubbs.test(DaysMVregEGFctrl)           # p-value = 0.412
```

```
# then, after elimination of putative outliers and related data in paired samples:

grubbs.test(AreaTot_d0_VEGF)            # p-value = 0.3478
grubbs.test(AreaTot_d0_VEGFctrl)        # p-value = 0.7749
grubbs.test(AreaReg_d3_VEGF)            # p-value = 0.1202
grubbs.test(AreaReg_d3_VEGFctrl)        # p-value = 0.2303
grubbs.test(AreaReg_d3_EGF)             # p-value = 0.07042
grubbs.test(AreaReg_d3_EGFctrl)         # p-value = 0.3637
grubbs.test(DaysMVregVEGF)              # p-value = 1
grubbs.test(DaysMVregVEGFctrl)          # p-value = 1
```

SUPPLEMENTARY DATA S5

# TEST PROCEDURE PERFORMED IN R FOR TESTING NORMALITY DISTRIBUTION OF SAMPLES
## STARTING FROM DATASET IN SUPPLEMENTARY DATA S3
## (*): p-value < or = 0.05

```
library(nortest)
                 # colony size after ablation

                            # VEGF
shapiro.test(AreaTot_d0_VEGF)           # p-value = 0.353
shapiro.test(AreaTot_d0_VEGFctrl)       # p-value = 0.0008032 (*)
                            # EGF
shapiro.test(AreaTot_d0_EGF)            # p-value = 0.289
shapiro.test(AreaTot_d0_EGFctrl)        # p-value = 0.01581 (*)


                 # regenerated area after 3 days

                            # VEGF
shapiro.test(AreaReg_d3_VEGF)           # p-value = 0.0004474 (*)
shapiro.test(AreaReg_d3_VEGFctrl)       # p-value = 0.00412   (*)
                            # EGF
shapiro.test(AreaReg_d3_EGF)            # p-value = 0.06445
shapiro.test(AreaReg_d3_EGFctrl)        # p-value = 0.008645 (*)


                 # ablated region (lateral cut edges distance)

                            # VEGF
shapiro.test(DistLCedges_d0_VEGF)       # p-value = 0.05445
shapiro.test(DistLCedges_d0_VEGFctrl)   # p-value = 0.2877
                            # EGF
shapiro.test(DistLCedges_d0_EGF)        # p-value = 0.3318
shapiro.test(DistLCedges_d0_EGFctrl)    # p-value = 0.2361


                 # regenerated marginal vessels (days)

                            # VEGF
shapiro.test(DaysMVregVEGF)             # p-value = 0.006088  (*)
shapiro.test(DaysMVregVEGFctrl)         # p-value = 7.552e-05 (*)
```

```
                                # EGF
shapiro.test(DaysMVregEGF)              # p-value = 0.001826 (*)
shapiro.test(DaysMVregEGFctrl)          # p-value = 0.005939 (*)



                ## TEST PROCEDURE ON LOG10 TRANSFORMED SAMPLES

      shapiro.test(log10_AreaTot_d0_VEGF)     # p-value = 0.9791
      shapiro.test(log10_AreaTot_d0_VEGFctrl)# p-value = 0.1949

      shapiro.test(log10_AreaTot_d0_EGF)      # p-value = 0.7812
      shapiro.test(log10_AreaTot_d0_VEGFctrl)# p-value = 0.1949

      shapiro.test(log10_AreaReg_d3_VEGF)     # p-value = 0.9878
      shapiro.test(log10_AreaReg_d3_VEGFctrl)# p-value = 0.0716

      shapiro.test(log10_AreaReg_d3_EGF)      # p-value = 0.4673
      shapiro.test(log10_AreaReg_d3_EGFctrl) # p-value = 0.01909 (*)

      shapiro.test(log10_DaysMVregVEGF)       # p-value = 0.001249 (*)
      shapiro.test(log10_DaysMVregVEGFctrl)   # p-value = 1.721e-05(*)

      shapiro.test(log10_DaysMVregEGF)        # p-value = 0.005505 (*)
      shapiro.test(log10_DaysMVregEGFctrl)    # p-value = 0.006891 (*)
```

```
#  SUPPLEMENTARY DATA S6

#  LEVENE'S TEST PERFORMED IN R FOR HOMOGENEITY OF VARIANCE
## ACROSS NORMALLY DISTRIBUTED PAIRED SAMPLES
## (*): p-value < or = 0.05

library(car)
                             ## colony size after ablation

                                   # VEGF
# not performed - normality assumption failed

                                   # EGF
# not performed - normality assumption failed



                             ## regenerated area

                                   # VEGF
# not performed - normality assumption failed

                                   # EGF
# not performed - normality assumption failed



                             ## ablated region (lateral cut edges distance)

                                   # VEGF
data <- c(DistLCedges_d0_VEGF,DistLCedges_d0_VEGFctrl)
groups = factor(rep(letters[1:2], times=c(length(DistLCedges_d0_VEGF),length(DistLCedges_d0_VEGFctrl))))

leveneTest(data, groups)    # p-value = 0.1864

                                   # EGF
data <- c(DistLCedges_d0_EGF,DistLCedges_d0_EGFctrl)
groups = factor(rep(letters[1:2], times=c(length(DistLCedges_d0_EGF),length(DistLCedges_d0_EGFctrl))))

leveneTest(data, groups)    # p-value = 0.1036
```

```
                        ## regenerated marginal vessels (days)

                                  # VEGF
# not performed - normality assumption failed

                                  # EGF
# not performed - normality assumption failed




     # TEST PROCEDURE ON LOG10 TRANSFORMED SAMPLES

                            ## colony size after ablation

                                  # VEGF
     data <- c(log10_AreaTot_d0_VEGF,log10_AreaTot_d0_VEGFctrl)
     groups = factor(rep(letters[1:2], times=c(length(log10_AreaTot_d0_VEGF),length(log10_AreaTot_d0_VEGFctrl))))

     leveneTest(data, groups)    # p-value = 0.7061

                                  # EGF
     data <- c(log10_AreaTot_d0_EGF,log10_AreaTot_d0_EGFctrl)
     groups = factor(rep(letters[1:2], times=c(length(log10_AreaTot_d0_EGF),length(log10_AreaTot_d0_EGFctrl))))

     leveneTest(data, groups)    # p-value = 0.6021


                            ## regenerated area

                                  # VEGF
     data <- c(log10_AreaReg_d3_VEGF,log10_AreaReg_d3_VEGFctrl)
     groups = factor(rep(letters[1:2], times=c(length(log10_AreaReg_d3_VEGF),length(log10_AreaReg_d3_VEGFctrl))))

     leveneTest(data, groups)    # p-value = 0.01935 (*)

                                  # EGF
     # not performed - normality assumption failed
```

regenerated marginal vessels (days)

VEGF

not performed - normality assumption failed

EGF

not performed - normality assumption failed

SUPPLEMENTARY DATA S7

# PROCEDURE USED IN R TO PLOT INTERVALS OF THE MEANS (0.95% CONFIDENCE LIMITS)
## OF STARTING CONDITIONS FOR PAIRED SAMPLES

```
library(psych)

                              ## colony size after ablation (TEST PROCEDURE ON LOG10 TRANSFORMED SAMPLES)


                                    # VEGF
      data<- c(log10_AreaTot_d0_VEGF, log10_AreaTot_d0_VEGFctrl)
      gruppi = factor(rep(letters[1:2], times=c(length(log10_AreaTot_d0_VEGF),length(log10_AreaTot_d0_VEGFctrl))))
      error.bars.by(data,gruppi,by.var=T)

                                    # EGF
      data<- c(log10_AreaTot_d0_EGF, log10_AreaTot_d0_EGFctrl)
      gruppi = factor(rep(letters[1:2], times=c(length(log10_AreaTot_d0_EGF),length(log10_AreaTot_d0_EGFctrl))))
      error.bars.by(data,gruppi,by.var=T)


                        ## ablated region (lateral cut edges distance)

                              # VEGF
data <- c(DistLCedges_d0_VEGF,DistLCedges_d0_VEGFctrl)
gruppi = factor(rep(letters[1:2], times=c(length(DistLCedges_d0_VEGF),length(DistLCedges_d0_VEGFctrl))))
error.bars.by(data,gruppi,by.var=T)

                              # EGF
data <- c(DistLCedges_d0_EGF,DistLCedges_d0_EGFctrl)
gruppi = factor(rep(letters[1:2], times=c(length(DistLCedges_d0_EGF),length(DistLCedges_d0_EGFctrl))))
error.bars.by(data,gruppi,by.var=T)
```

SUPPLEMENTARY TABLE S8.

Description of samples used for correlation analyses, which are in Supplementary Data S9.

**DESCRIPTION OF SAMPLES** ***(i.e., collected measures from all colonies injected with PBS)***	**Name of the sample (as named in Supplementary Data S9)**	**Samples described in Table 1 which data have been aggregated**	**related samples for each correlation analysis (and the figure showing result)**
data related to starting condition in term of ***colony size***	AllCtrlsAreaTotD0	AreaTot_d0_VEGFctrl + AreaTot_d0_EGFctrl	● regenerated area (Fig. 6 A) ● time of marginal vessel regeneration (Fig. 6 C)
data related to starting condition in term of ***ablated region***	AllCtrlsDistLCedgesD0	DistLCedges_d0_VEGFctrl + DistLCedges_d0_EGFctrl	● regenerated area (Fig. 6 B) ● time of marginal vessel regeneration (Fig. 6 D)
data related to CCS regeneration in term of ***regenerated areas***	AllCtrlsAreaTotD3	AreaReg_d3_VEGFctrl + AreaReg_d3_EGFctrl	● ablated region (Fig. 6 B) ● time of marginal vessel regeneration (Fig. 6 E)
data related to CCS regeneration in term of ***time of marginal vessel regeneration***	AllCtrlsDaysMVreg	DaysMVregVEGFctrl + DaysMVregEGFctrl	● ablated region (Fig. 6 D) ● regenerated area (Fig. 6 E)

SUPPLEMENTARY DATA S9

DATASET TO IMPORT THE AGGREGATED CONTROL SAMPLES IN R
AND TO OBTAIN, FOLLOWING THE INSTRUCTION IN THE
SUPPLEMENTARY DATA S10, THE REGRESSION PLOTS AND THE
COEFFICIENTS OF DETERMINATION (R^2) OF THE REGRESSION.

```
#1______________________
                    ## colony size VERSUS regenerated area.

              # Samples

AllCtrlsAreaTotD0 <- scan()
35531054 37505911 35566298 14886998 25011034
15476291 30866356 23251402 17772963 36006383
28329622 35527648 24131725 14194599 42262730
79812606 28833941 30521187 28737823
14846368 17941039 15978138 13163135 23746737
14686625 15411596 24247940 19528538 42121133
28276128 20481610

AllCtrlsAreaRegD3  <- scan()
  19885   1167173   -22465   -82765  3258803
  84775   3382075   390894    42390   342345
1253436    984490    84538  2211244  2886969
2817288    335158  1881259   340019
 922096   1513287  2776660  2464037  1971225
  38440    441013   888858  1142205  6499956
1853451   1931061


#2______________________
                    ## ablated region VERSUS regenerated area.

              # Samples

AllCtrlsDistLCedgesD0 <- scan()
3723 4522 4328 3228 4802
4104 4228 3563 3136 4533
4480 4908 3413 3045 3550
4316 4093 4089 4624
4474 5648 4900 3556 3146
5353 4731 4188 3372 5630
```

```
4925 3259


AllCtrlsAreaRegD3 <- scan()
  19885   1167173   -22465   -82765  3258803
  84775   3382075   390894    42390   342345
1253436    984490    84538  2211244  2886969
2817288    335158  1881259   340019
 922096   1513287  2776660  2464037  1971225
  38440    441013   888858  1142205  6499956
1853451   1931061



#3______________________
                 ## ablated region VERSUS marginal vessel regeneration.

           # Samples

AllCtrlsDistLCedgesD0 <- scan()
3723 4522 4328 3228 4802
4104 4228 3563 3136 4533
4480 4908 3413 3045 3550
4316 4093 4089 4624
4474 5648 4900 3556 3146
5353 4731 4188 3372 5630
4925 3259

AllCtrlsDaysMVreg <- scan()
5 5 4 5 4
4 5 5 5 5
4 5 3 5 4
2 5 5 4
5 5 5 2 3
5 3 4 5 5
3 3
```

```
#4______________________
                 ## colony size VERSUS marginal vessel regeneration.

           # Samples

AllCtrlsAreaTotD0 <- scan()
35531054 37505911 35566298 14886998 25011034
15476291 30866356 23251402 17772963 36006383
28329622 35527648 24131725 14194599 42262730
79812606 28833941 30521187 28737823
14846368 17941039 15978138 13163135 23746737
14686625 15411596 24247940 19528538 42121133
28276128 20481610

AllCtrlsDaysMVreg <- scan()
5 5 4 5 4
4 5 5 5 5
4 5 3 5 4
2 5 5 4
5 5 5 2 3
5 3 4 5 5
3 3



#5______________________
                 ## regenerated area VERSUS marginal vessel regeneration.

           # Samples

AllCtrlsAreaRegD3 <- scan()
  19885   1167173   -22465   -82765  3258803
  84775   3382075   390894    42390   342345
1253436    984490    84538  2211244  2886969
2817288    335158  1881259   340019
 922096   1513287  2776660  2464037  1971225
  38440    441013   888858  1142205  6499956
1853451   1931061
```

```
AllCtrlsDaysMVreg <- scan()
5 5 4 5 4
4 5 5 5 5
4 5 3 5 4
2 5 5 4
5 5 5 2 3
5 3 4 5 5
3 3
```

SUPPLEMENTARY DATA S10

# PROCEDURE PERFORMED IN R TO OBTAIN
## THE REGRESSION PLOTS AND THE
## COEFFICIENTS OF DETERMINATION R^2 OF THE REGRESSION,
## STARTING FROM DATASET IN SUPPLEMENTARY DATA S9:

```
library(nsRFA)


#1______________________
                 ## colony size VERSUS regenerated area.


           # R^2 and regression plot:

AllCtrlsAreaTotD0_vs_AllCtrlsAreaRegD3 <- lm(AllCtrlsAreaTotD0 ~ AllCtrlsAreaRegD3)
R2.lm (AllCtrlsAreaTotD0_vs_AllCtrlsAreaRegD3)
                                  # R^2 = 0.1021256

plot(AllCtrlsAreaTotD0 ~ AllCtrlsAreaRegD3)




#2______________________
                 ## ablated region VERSUS regenerated area.


           # R^2 and regression plot:

AllCtrlsDistLCedgesD0_vs_AllCtrlsAreaRegD3 <- lm(AllCtrlsDistLCedgesD0 ~ AllCtrlsAreaRegD3)
R2.lm (AllCtrlsDistLCedgesD0_vs_AllCtrlsAreaRegD3)
                                  # R^2 = 0.07001780

plot(AllCtrlsDistLCedgesD0 ~ AllCtrlsAreaRegD3)
```

```
#3______________________
                 ## ablated region VERSUS marginal vessel regeneration.


           # R^2 and regression plot:

AllCtrlsDistLCedgesD0_vs_AllCtrlsDaysMVreg <- lm(AllCtrlsDistLCedgesD0 ~ AllCtrlsDaysMVreg)
R2.lm (AllCtrlsDistLCedgesD0_vs_AllCtrlsDaysMVreg)
                                  # R^2 = 0.030048624

plot(AllCtrlsDistLCedgesD0 ~ AllCtrlsDaysMVreg)




#4______________________
                 ## colony size VERSUS marginal vessel regeneration.


           # R^2 and regression plot:

AllCtrlsAreaTotD0_vs_AllCtrlsDaysMVreg <- lm(AllCtrlsAreaTotD0 ~ AllCtrlsDaysMVreg)
R2.lm (AllCtrlsAreaTotD0_vs_AllCtrlsDaysMVreg)
                                  # R^2 = 0.04789299

plot(AllCtrlsAreaTotD0 ~ AllCtrlsDaysMVreg)




#5______________________
                 ## regenerated area VERSUS marginal vessel regeneration.


           # R^2 and regression plot:

AllCtrlsAreaRegD3_vs_AllCtrlsDaysMVreg <- lm(AllCtrlsAreaRegD3 ~ AllCtrlsDaysMVreg)
R2.lm (AllCtrlsAreaRegD3_vs_AllCtrlsDaysMVreg)
                                  # R^2 = 0.01218851

plot(AllCtrlsAreaRegD3 ~ AllCtrlsDaysMVreg)
```

SUPPLEMENTARY DATA S11

# PROCEDURE TO OBTAIN THE BOXPLOTS OF THE
## SAMPLES RELATED TO THE REGENERATION OF THE CCS
## STARTING FROM DATASET IN SUPPLEMENTARY DATA S3

```
# regenerated area after 3 days

# VEGF (LOG10 TRANSFORMED SAMPLES)
boxplot(log10_AreaReg_d3_VEGF,log10_AreaReg_d3_VEGFctrl)

# EGF
boxplot(AreaReg_d3_EGF,AreaReg_d3_EGFctrl)


# regenerated marginal vessels (days)

# VEGF
boxplot(DaysMVregVEGF,DaysMVregVEGFctrl)

# EGF
boxplot(DaysMVregEGF,DaysMVregEGFctrl)
```

#____

# ANALYSES PERFORMED IN R TO TEST IF THE MEANS OF THE PAIRED SAMPLES
## RELATED TO REGENERATION OF THE CCS ARE EQUAL
## STARTING FROM DATASET IN IN SUPPLEMENTARY DATA S3
## (*): p-value < or = 0.05

```
#  PAIRED T TEST (PARAMATRIC) (ACROSS NORMALLY DISTRIBUTED
## PAIRED SAMPLES WITH HOMOGENEITY OF VARIANCE)

# NO ONE
```

```
# WILCOXON SIGNED-RANK TEST (NON PARAMETRIC)

# regenerated area after 3 days

# VEGF (LOG10 TRANSFORMED SAMPLES)
wilcox.test(log10_AreaReg_d3_VEGF, log10_AreaReg_d3_VEGFctrl, exact= FALSE, paired = TRUE, correct = FALSE)
# p-value = 0.01295 (*)

# EGF
wilcox.test(AreaReg_d3_EGF, AreaReg_d3_EGFctrl, exact= FALSE, paired = TRUE, correct = FALSE)
# p-value = 0.002218 (*)


# marginal vessel regeneration (days)

# VEGF
wilcox.test(DaysMVregVEGF, DaysMVregVEGFctrl, exact= FALSE, paired = TRUE, correct = FALSE)
# p-value = 0.0005109 (*)

# EGF
wilcox.test(DaysMVregEGF, DaysMVregEGFctrl, exact= FALSE, paired = TRUE, correct = FALSE)
# p-value = 0.009339 (*)
```
